# A Molecular Dynamics Approach to the Impacts of Oxidative Aging on the Engineering Characteristics of Asphalt

**DOI:** 10.3390/polym14142916

**Published:** 2022-07-19

**Authors:** Wei Cao, Elham Fini

**Affiliations:** 1 School of Civil Engineering, Central South University, Changsha 410075, China; 2 School of Sustainable Engineering and the Built Environment, Arizona State University, Tempe, AZ 85287, USA; efini@asu.edu

**Keywords:** oxidative aging, diffusion rate, asphalt–aggregate adhesion, molecular polarity, electrostatic potential surface

## Abstract

Oxidative aging is an inevitable environmental factor that accelerates asphalt pavement deterioration. This study employed a molecular dynamics simulation to investigate the impact of aging on asphalt cement from the perspectives of thermodynamic properties, and diffusion and adhesion characteristics. Results indicate that aging increased bulk density from 1.008 to 1.081 g/cm^3^ and cohesive energy density by 15.6%, which was attributed to the promoted molecular polarity and intermolecular attractiveness. The enhanced molecular interactions also reduced molecular mobility, which led to an increase in the glass transition temperature by 30 K, suggesting that aging diminished the resistance of asphalt to thermal cracking. Simulations of the diffusion behaviors across different temperatures demonstrated that the Arrhenius relationship described well the temperature dependence of the diffusion coefficient, and that aging considerably slowed down the diffusion process as represented by Arrhenius prefactor *D*_0_, which dropped by 38.2%. The asphalt–aggregate adhesion was assessed using layered models with and without a water interlayer of different thicknesses. The adhesion was enhanced upon aging due to the significantly improved electrostatic interactions at the interface. Evaluation of the residual adhesion with the presence of interfacial water suggested that aging would raise the moisture susceptibility of asphalt pavement. The increase in molecular polarity was considered to be highly responsible for these aging consequences, and was thus further investigated via the electrostatic potential surface and dipole moment.

## 1. Introduction

As a byproduct of crude-oil distillation, asphalt has been most widely used in pavement engineering. It plays a central role in maintaining the material integrity and strength of pavement materials against traffic loading and environmental weathering. Despite ubiquity and macroscopic homogeneity, the chemical composition of asphalt still remains a mystery, as it comprises numerous molecules of different elements, structures, and properties. These molecules are usually categorized and separated as per solubility into the four fractions of saturates, aromatics, resins, and asphaltenes (i.e., the so-called SARA fractionation). There is a continuing debate on the representation of the internal structure of asphalt. Two philosophies primarily exist: one considers asphalt to have a colloidal structure, whereas the other views it as a homogeneous liquid without colloidal characteristics [1]. Nevertheless, to a great extent, asphalt can be practically treated as a mixture of polymers, as many concepts (e.g., glass transition temperature, molecular weight distribution, and complex modulus) and modeling theories (such as viscoelasticity and free volume theory) that are largely rooted in polymer materials have been widely applied to characterize asphalt, and are used in the inference of engineering performance [2,3,4].

As an organic binding agent, asphalt cement is prone to oxidative aging, which leads to degradation in engineering properties such as stress relaxation and healing, thereby promoting pavement cracking [5,6]. Petersen et al. [7,8,9] conducted a wealth of in-depth investigations into asphalt aging, and proposed a dual oxidation mechanism that consisted of an initial fast reaction followed by a slow and long-term reaction. The initial spurt was characterized by the formation of sulfoxides due to the oxidation of highly reactive hydrocarbons to produce free radicals that reacted with alkyl and aryl sulfides. The second stage featured the formation of ketones and alcohols due to oxidation at the benzyl carbons. During both reactions, the introduced polar functional groups increased the molecular polarity and were thus expected to enhance the intermolecular forces, which was responsible for the increased stiffness and viscosity. The formation of ketones and sulfoxides as major oxidation products was also confirmed by quantum-chemistry-based simulations [10].

In theory, the microstructural characteristics (molecular composition and interactions) of asphalt should dictate its engineering properties and behaviors, and yet the exact mechanisms linking the two length scales still remain largely unknown. Among the various efforts devoted to this, the molecular dynamics (MD) simulation stands out as a viable and versatile approach that has been used to interpret the thermodynamic properties and mechanical behaviors of asphalt. Li and Greenfield [11,12] synthesized the existing findings and proposed a set of 12 molecules representing the four fractions of asphalt. They demonstrated that this asphalt system was able to provide more realistic predictions of density, viscosity, and relaxation time as compared to previous models. Pan and Tarefder [13] utilized this model to evaluate the impacts of aging on the bulk and mechanical properties of asphalt. Aging had a considerable impact on the intermolecular energy by increasing the electrostatic contribution, while the van der Waals (vdW) component was almost unaffected. Enhancement in the electrostatic attractiveness was attributed to the polar groups added onto the oxidized asphalt molecules, and its correlation was established with the improved hardness of the asphalt model, as noted in the simulated mechanical responses. The aging-induced stronger molecular interactions was also reflected through increase in density and cohesive energy density (CED), as well as reduced diffusion coefficient in a number of studies using different asphalt models [14,15,16,17].

The MD approach to the adhesion characteristics of asphalt has been limited to the chemical aspects of the contacting surfaces, and it provides valuable insights into pavement distress such as raveling and stripping [18,19]. Gao et al. [15] compared four different minerals and found that, when the substrate varied from acidic to alkaline, the adhesion energy increased, and the nature of the primary contribution to adhesion changed from vdW to electrostatic. Asphalt aging was generally reported to yield higher adhesion energies, but also more debonding work with the presence of water [20,21]. Such a complexity corresponds to the mixed experimental findings regarding the effect of aging on the adhesion performance between asphalt and mineral aggregate [22].

The existing literature mostly addresses the impacts of aging on a few properties without providing sufficient insights into their correlations with the engineering performance of asphalt. The present study investigates the effects of oxidative aging regarding fundamental thermodynamic properties, and diffusion and adhesion characteristics. Further, the role of increased molecular polarity was highlighted in discussing the aging-induced consequences. The findings add to the literature in understanding material properties and engineering performance from slightly different perspectives in terms of data analysis and presentation.

## 2. Material Models and Simulation Method

The MD simulation was performed using commercial software Materials Studio [23]. The Condensed-phase Optimized Molecular Potentials for Atomistic Simulation Studies (COMPASS) II forcefield was employed to describe the molecular interactions. The parameters in this forcefield were derived through a combination of fits to the ab initio data for valence parameters, and experimental data for nonbond parameters [24]. For vdW interactions, the atom-based summation method was used with a cut-off distance of 15.5 Å, and the long-range correction was applied beyond this range. For electrostatic interactions, the Ewald summation was adopted. All the computations were performed with a time step of 1.0 fs.

### 2.1. Asphalt Bulk Models

**Figure 1:**
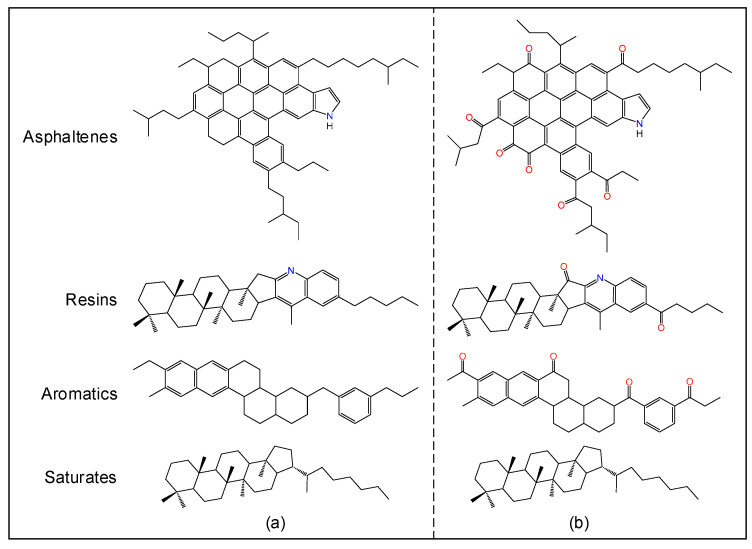
Molecular structures representing the four fractions of the (**a**) virgin and (**b**) aged asphalts.

Asphalt is an extremely complex mixture of hydrocarbons for which the simulation is usually carried out using a set of molecular models representative of the different fractions. Given considerations on the computational cost, the virgin (unaged) asphalt system in this study was established using one molecular structure for each of the four fractions on the basis of Li and Greenfield’s model [11]. Specifically, the virgin system consisted of asphaltene–pyrrole, quinolinohopane, perhydrophenanthrene–naphthalene (PHPN), and hopane at a number ratio of 2:6:8:2, as shown in Figure 1a and Table 1.

As previously mentioned, ketones and sulfoxides are the major oxidation products occurring at the benzyl carbons and alkyl/aryl sulfides, respectively. Therefore, the structures for the virgin asphaltene, resin, and aromatic fractions were modified accordingly to represent the oxidized counterparts (Figure 1b) [17]. The nonpolar saturate fraction rarely changes with time [25]; thus, the same structure was retained. In the aged model, the fraction ratio was slightly adjusted to account for the component shift typically observed during the asphalt oxidation process [5,26]. Table 1 provides a compositional summary for the virgin and aged asphalt models.

### 2.2. Mineral Substrate

**Figure 2:**
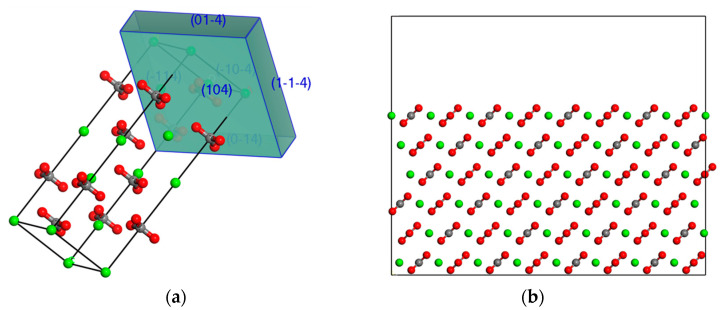
Preparation of calcite substrate: (**a**) habit faces identified by the attachment energy theory; (**b**) supercell substrate (with a vacuum slab inserted on top).

Calcite (commonly found in limestone) was used as the mineral substate to evaluate the adhesion properties of the virgin and aged asphalts. The stone aggregates used in asphalt mixtures are obtained mainly through a mechanical crushing process during which the mineral tends to rupture along the surfaces where interlayer bonding strengths are weak. For this reason, the crystal first needs to be cleaved to expose such surfaces using the Morphology module in Materials Studio. Figure 2a depicts the morphology calculation result based on attachment energy theory [27]. This theory considers attachment energy as a measure of crystal growth rate normal to a face, and that the face with higher attachment energy would grow faster and thus be less morphologically important. The use of this theory resulted in a single family of lattice planes that were represented in terms of Miller indices as (1 0 4). This family of planes was also identified using the Bravais–Friedel–Donnay–Harker (BFDH) approach [28], but with the second highest morphological importance (not shown). BFDH theory only considers the lattice geometry in computation without taking into account the details such as chemical nature and molecular packing in the crystal.

Considering additional experimental evidence confirming that surface (1 0 4) is the most commonly exposed plane for calcite [29,30], this habit face was used for crystal cleavage in this study. Supercells with appropriate dimensions depending on the need (discussed later) were then constructed for the interface models. A fractional thickness of 6 was applied, and the resulting supercell substrates had a thickness of 17 Å (greater than the vdW cut-off distance), as illustrated in Figure 2b.

### 2.3. General Setup for the MD Simulation

For constructing the bulk model of asphalt, the Amorphous Cell (AC) module was utilized, which allowed for creating cells with periodical boundary conditions containing random arrangements of the model molecules. The initial density was set at 0.6 g/cm^3^ (lower than the equilibrium level) to encourage the randomness in generating the asphalt AC configurations.

**Figure 3:**
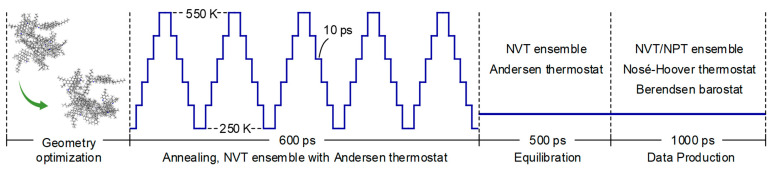
Four-step process employed to bring the system to equilibrium with physically sound dynamics (time not to scale).

In this study, unless otherwise stated, the completion of the model setup was followed by a four-step process, as illustrated in Figure 3, to reach the final state at equilibrium that is ready for analysis.

- Step 1: geometry optimization to eliminate the unrealistic molecular overlapping and adjust unstable high-energy configurations.
- Step 2: annealing to overcome energy barriers by periodically heating and cooling the system (250 to 550 K) to find the energetically favorable minima using the canonical ensemble NVT (a constant number of atoms N, a constant volume V, and a controlled temperature T).
- Step 3: equilibration to bring the system to equilibration over a simulation time of 500 ps using the NVT ensemble with an Andersen thermostat.
- Step 4: data production run to further equilibrate the system under the NVT or NPT ensemble (a constant number of atoms N, a controlled pressure P, and a controlled temperature T) depending on the model of interest, using the Nosé–Hoover thermostat (for physically sound dynamics) and Berendsen barostat, over a simulation time of 1 ns.

## 3. Results and Discussion

### 3.1. Bulk Thermodynamic Properties

This section presents the thermodynamic properties of the virgin and aged bulk asphalts predicted from the MD simulation, including density, cohesive energy density (CED), and glass transition temperature *T_g_*. Results preliminarily validated the established molecular model and the MD simulation process by comparing the existing experimental and simulation findings.

#### 3.1.1. Prediction of Density

The four-component asphalt models were brought to equilibrium under atmospheric pressure (1 atm) and room temperature (298.15 K) using the four-step simulation process (NPT for the last step). The final density for the virgin asphalt was equilibrated at 1.008 g/cm^3^, which agreed well with the existing simulation results and was comparable with experimental values in the range from 1.0 to 1.04 g/cm^3^ [11,17,20]. The aged model yielded a slightly higher density of 1.081 g/cm^3^. The increase in density could be partly attributed to the fact that oxidation increased the molecular weight of the polar fractions by introducing oxygen atoms and tilted the composition balance towards asphaltenes, which was reflected in the molecular structures and proportions used in simulation.

#### 3.1.2. Prediction of Cohesive Energy Density

The CED of a material in a condensed state is defined as the amount of energy required to completely remove a unit volume of molecules against all attractive forces from the neighboring molecules. Hence, it can be used as an overall measure of the strength of intermolecular interactions. The predicted CED values for the virgin and aged models (eventually equilibrated under 1 atm) were 3.02 × 10^8^ and 3.49 × 10^8^ J/m^3^, respectively. The primary contribution to CED was vdW interactions, and aging slightly reduced the vdW contribution from 96.7% to 91.9% due to the increased molecular polarity. These observations agree with values reported in the literature [15,31].

#### 3.1.3. Prediction of Glass Transition Temperature

Asphalt exhibits a glassy state when the temperature is below *T_g_*, and rubbery behavior above *T_g_*. The glass transition temperature can be thermodynamically interpreted as the threshold below which the reduction in free volume becomes significantly slow over time as temperature decreases [32]. The physical hardening phenomenon (stiffness increase over time) of asphalt occurs at temperatures around *T_g_* [33]; more importantly, this parameter is closely related to the propensity of asphalt pavement for thermal cracking [34].

The glass transition temperature is commonly determined as the temperature at which the two asymptotes to the glassy and rubbery regions intersect in the space of specific volume versus temperature. During the simulation process, a trajectory file was first created that contained three different AC configurations as replicates for the purpose of minimizing the potential scattering in *T_g_* due to AC randomness. A *Perl* script was prepared that iterated over every configuration, executing the following algorithm: a short NVT annealing followed by NPT equilibration runs at decreasing temperatures from 600 to 100 K with a step size of 2 K. At each temperature step, the density was checked every 10 ps, and the equilibration dynamics run was performed until the density had stabilized within a tolerance of 0.02 g/cm^3^. Then, a further NPT production run over 10 ps was used to obtain the final density data before entering the next temperature step.

**Figure 4:**
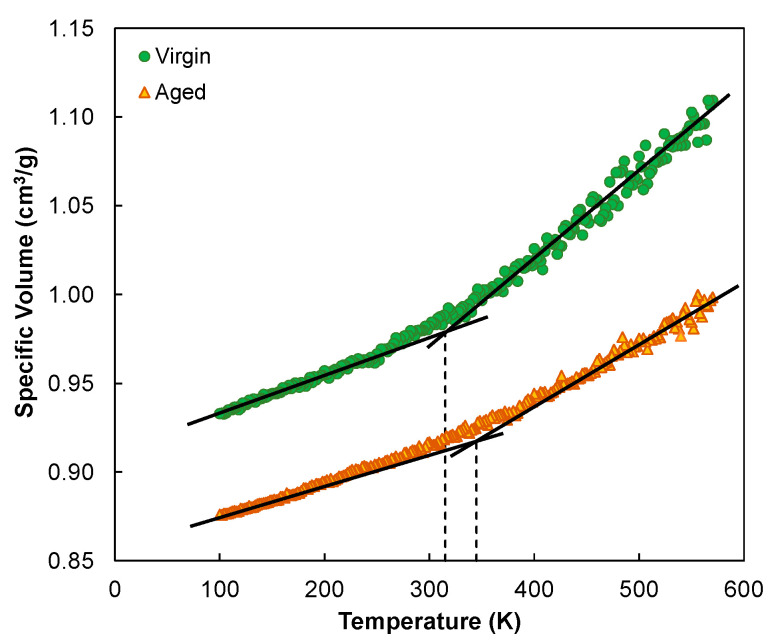
Determination of the glass transition temperatures.

Figure 4 presents the simulation data of averaged specific volume versus temperature and *T_g_* determination for the virgin and aged asphalts. The virgin system yielded a glass transition temperature of 315 K, which was in line with the typical values reported from existing simulations [20,35]. Compared to the experimental range of 223 to 303 K [32], this *T_g_* result was slightly higher, which could be partly attributed to the higher cooling rate used in simulation [36]. The aged asphalt model provided an increased *T_g_* value of 345 K, as confirmed by earlier experimental findings [37]. In addition, by comparing the change in the slope of the two asymptotes, the reduction in the free volume of the aged asphalt exhibited an overall lower sensitivity to temperature drop. This observation can be ascribed to the stronger intermolecular attractiveness that restricted the molecular mobility in the aged system.

### 3.2. Asphalt Diffusion Characteristics

**Figure 5:**
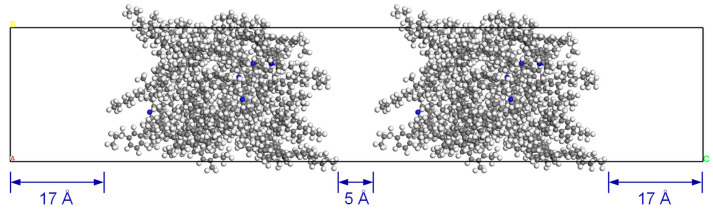
Geometry of the diffusion model.

The potential of asphalt diffusion was evaluated in a setup that simulated the crack healing process [38]. As cracking in reality occurs well after the system reaches thermodynamic equilibrium, the equilibrated AC configuration was used to build the diffusion model as opposed to the use of an unequilibrated system in the literature [39]. Specifically, the two models of the virgin or aged asphalt were arranged in a simulation cell with a 5 Å vacuum space in between to represent the crack, as shown in Figure 5. Meanwhile, at the two ends, a 17 Å thick vacuum slab was applied in order to eliminate interference from the mirror images of the model. Afterwards, the MD simulation was performed using the NPT ensemble at 1 atm for a duration of 1 ns to inspect the diffusion process.

The diffusion process was measured by the commonly used concept of mean square displacement (MSD), which describes the translational mobility of particles over time. It can be perceived as the spatial extent that is “explored” by the randomly moving molecules due to diffusion. The MSD as a function of time is defined as

(1) $$\mathrm{MSD}\left(t\right)=\frac{1}{N}\overset{N}{\underset{i=1}{\sum }}{\left|r_{i}\left(t\right)-r_{i}\left(0\right)\right|}^{2}$$

where ***r****_i_*(*t*) is the position vector of the *i*-th particle at time *t*, ***r****_i_*(0) denotes the reference position at time 0, and *N* is the total number of particles to be averaged.

**Figure 6:**
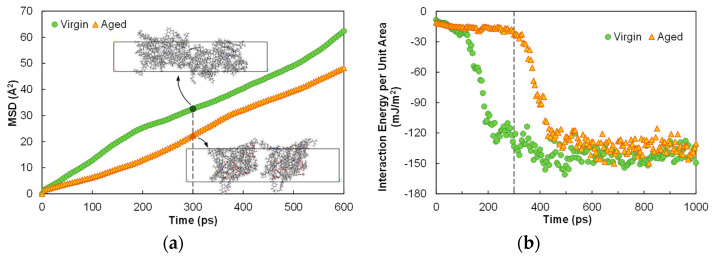
Comparison of the diffusion processes at the temperature of 298.15 K via the time histories of (**a**) MSD and (**b**) interaction energy (negative energy values indicating attractive interactions).

Figure 6a presents the time histories of MSD for the virgin and aged asphalts at the room temperature of 298.15 K. The virgin system exhibited higher molecular mobility than that of the aged system throughout the whole process. Specifically, at the simulation time of 300 ps, the two models in the virgin system already diffused across the crack space, and the crack was morphologically healed. The two models in the aged system, however, were still in the process of approaching each other with a noticeable void space in between.

Another perspective to examine the diffusion rate relied on the use of interaction energy between the two models in the cell. To account for the difference and variation in the cell dimensions, the interaction energy was further divided by the cross-sectional area of the cell, and results are compared in Figure 6b. The negative energies at the initial states of both systems suggested that the subsequent diffusion to heal the crack was thermodynamically feasible. A simulation time of 1 ns was sufficient to stabilize the interaction energy for both systems at room temperature, and the aged asphalt reached the energy equilibrium again at a lower rate. At 300 ps, the interaction energy per unit area for the virgin system was around −130 mJ/m^2^, close to the equilibrated level, but the value for the aged system was not much different from the starting point. Existing findings indicated that, for individual molecules, the diffusion rate is dependent on the factors including molecular weight, shape, and polarity [40,41]. Herein, the lower mobility of the aged asphalt as an assembly of oxidized molecules can be attributed to the increase in molecular polarity and weight that promoted the attractive interactions.

**Figure 7:**
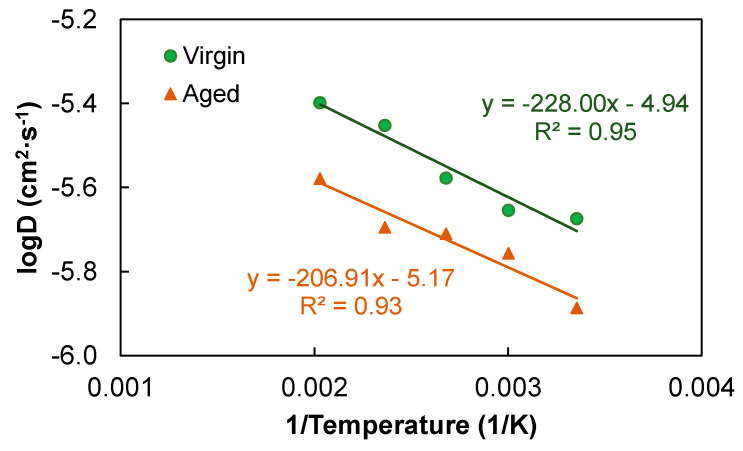
Relationships of the diffusion coefficient with temperature and the Arrhenius fits.

The MD simulation on the diffusion model was further performed at a number of higher temperatures: 333.15, 373.15, 423.15, and 493.15 K (i.e., 60, 100, 150, and 220 °C). The diffusion coefficient *D* for both the virgin and aged asphalts was obtained from the slope of the MSD plot (by a factor of 1/6), and its dependence on temperature was investigated. Figure 7 presents the relationships of *D* versus temperature and the fitting using the Arrhenius equation [12]:

(2) $$D=D_{0}e^{-E_{a}/RT}$$

where *E_a_* is the activation energy, *R* is the universal gas constant (8.314 J/mol/K), *T* is temperature, and *D*_0_ is the diffusion prefactor.

Figure 7 shows that, at all temperatures, the virgin system diffused at considerably higher rates than the aged system did, and the diffusion coefficients followed the Arrhenius behavior reasonably well. The slope of the fit in the space of log*D* versus 1/*T* was used to further obtain the activation energy, which was 4.4 and 4.0 kJ/mol for the virgin and aged asphalts, respectively. The virgin model yielded a higher activation energy for self-healing but the difference was marginal. The prefactor was 1.1 × 10^−5^ cm^2^/s for the virgin, considerably higher than the 6.8 × 10^−6^ cm^2^/s for the aged. Li and Greenfield [12] evaluated the correlations of activation energy and prefactor with respect to molecular weight. They reported that the activation energy for different asphalt molecules varied within a narrow range and had little dependence on the molecular weight, but the prefactor showed a clear decreasing trend with it. Considering the fact that the molecular weight increases with aging, prefactor *D*_0_ appeared to be a valid index representing the overall aging effect on the asphalt diffusion behavior. In addition, since the increased molecular polarity is also a pronounced aging outcome, further effort is worthwhile to examine its correlation with the diffusion parameters.

### 3.3. Asphalt–Aggregate Adhesion Characteristics

In this section, the asphalt–aggregate adhesion property is considered in both dry and wet conditions. The potential size effect was first investigated to determine the proper geometry of the mineral substrate used in simulation, for a balance between modeling accuracy and computational efficiency.

#### 3.3.1. Size Effect

Due to the randomness in generating the asphalt AC, different atoms and functional groups (especially the carbonyl in the aged case) appear at the asphalt–aggregate interface, which may affect the determination of adhesion energy. Statistically, this effect should diminish as the model size enlarges. For this reason, the size effect was first evaluated using the aged asphalt model to determine the proper dimension for the calcite substrate as previously shown in Figure 2b.

**Figure 8:**
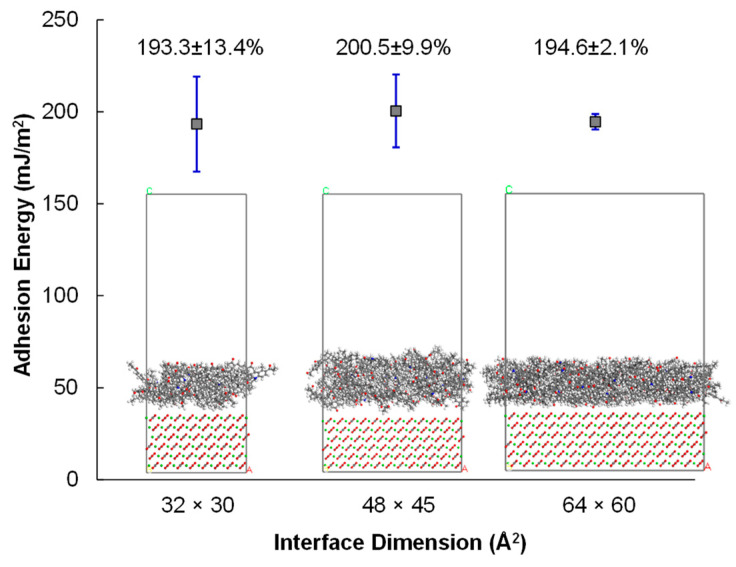
Size effect on the calculation of adhesion energy using the aged asphalt model.

In this process, the supercell substrate was first prepared with three different geometries: 32 × 30, 48 × 45, and 64 × 60 Å^2^, as indicated in Figure 8. For each geometry, four different asphalt AC configurations were constructed at the already-known equilibrium density by matching the interface geometry while maintaining the thickness as greater than the vdW cut-off distance. The interface model was created by fitting the asphalt AC onto the supercell substrate, while placing a 50 Å thick vacuum slab on top to avoid interference from the mirror images. Each of the 12 resulting interface models was then subjected to the four-step simulation process described earlier in Figure 3. In the substrate, the three bottom layers of atoms were fixed in their Cartesian coordinates, and the NVT ensemble was used in the final data production step.

The adhesion energy, described in Equation (3), was calculated for each scenario to obtain the mean and coefficient of variation (CoV), as included in Figure 8. The adhesion energy values were comparable to the experimental results reported in the literature [19,42]. The averaged adhesion energy appeared to not have been significantly affected by the three geometries selected for inspection. Nevertheless, variability due to AC randomness presented a decreasing trend according to CoV, as noted in earlier studies [43]. The substrate geometry of 64 × 60 Å^2^ yielded the lowest variability and was thus adopted for subsequent calculations.

#### 3.3.2. Dry Condition

**Figure 9:**
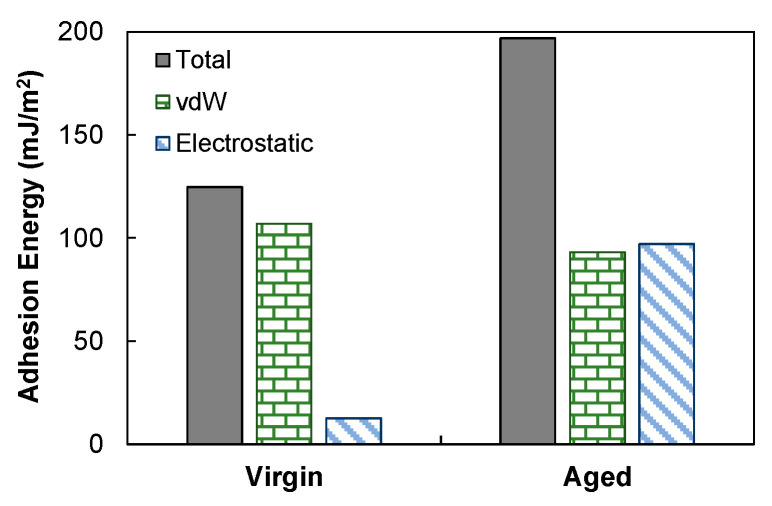
Total adhesion energies at the asphalt–calcite interface and the components.

The dry condition was evaluated to provide a baseline for the ensuing analysis involving the presence of water. In this case, the interface model consisted of the calcite supercell in the geometry of 64 × 60 Å^2^ and an AC for the virgin or aged asphalt. The adhesion energy for the asphalt–aggregate interface was determined in a way similar to the concept of interaction energy per unit area used previously in the discussion of diffusion. Specifically, the adhesion energy was calculated as

(3) $$E_{\mathrm{asph}-\mathrm{agg}}=\frac{E_{\mathrm{int}}}{A}=\frac{E_{\mathrm{interf}}-\left(E_{\mathrm{asph}}+E_{\mathrm{agg}}\right)}{A}$$

where *E*_asph-agg_ is adhesion energy, *A* is interface area, *E*_int_ is interaction energy, *E*_interf_ is the total potential energy of the whole interface model, and *E*_asph_ and *E*_agg_ are potential energies of the asphalt AC and mineral substrate, respectively. Negative values of *E*_asph-agg_ indicate attractive interactions. For facilitating data presentation, hereinafter the adhesion energies were given in terms of magnitude by dropping the negative sign, as shown in Figure 9.

Figure 9 also includes the vdW and electrostatic components of the total adhesion energies. Oxidation had a pronounced effect in improving the adhesion between asphalt and calcite. In the virgin case, the interface adhesion was primarily attributed to the vdW forces. After aging, the vdW contribution slightly dropped, but the electrostatic component was substantially increased to a level comparable to the vdW part. The raised importance of electrostatic contribution was ascribed to the interactions between the positively charged calcium at the mineral surface and the strong electronegativity of oxygen introduced into the aged asphalt. These observations were in line with general findings from experiments and simulations [15,44].

#### 3.3.3. Wet Condition

**Figure 10:**
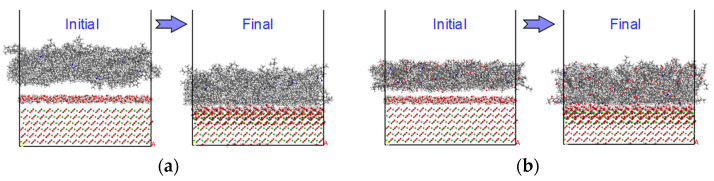
Initial and final model states considered for the wet condition with 3Å water layer at the interface. (**a**) Virgin; (**b**) aged (top vacuum slab not shown in its full thickness).

The wet condition was considered to be water that had penetrated into the interface and was present as an interlayer. The severity of moisture damage was simulated using four different water layer thicknesses: 3, 6, 9, and 12 Å, consisting of 400, 800, 1200, and 1600 water molecules, respectively. The previously detailed four-step process was applied to equilibrate the obtained three-layer interface models at the temperature of 298.15 K. Figure 10 shows the model states before and after equilibration for both the virgin and aged cases with a 3Å water interlayer.

**Figure 11:**
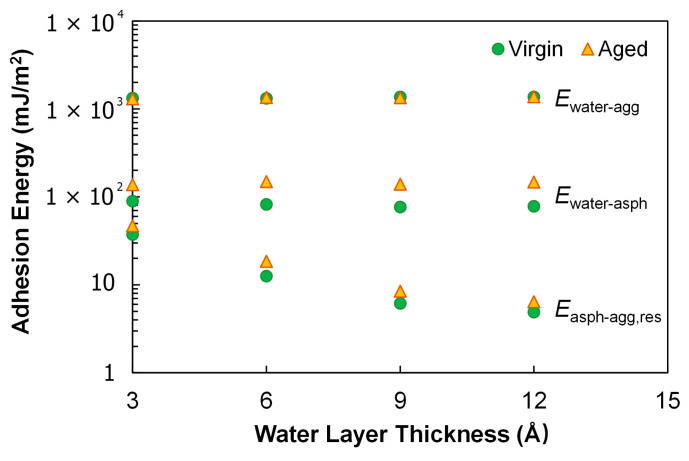
Adhesion energies for the three-layer interface model.

The adhesion energies of interest included the one between water and calcite *E*_water-agg_, and the one between water and asphalt *E*_water-asph_. In addition, despite the presence of water, there were still some weak interactions existing between asphalt and calcite, and this residual adhesion energy *E*_asph-agg,res_ was also calculated. Figure 11 shows these energy results as a function of the water layer thickness. Calcite is hydrophilic in nature, and *E*_water-agg_ was expectedly much higher than the remaining two in Figure 11; it was also an order of magnitude greater than the asphalt–calcite adhesion in the dry case as previously shown in Figure 9. The asphalt–calcite adhesion was significantly reduced with the presence of water, and diminished further with increase in the water layer thickness. Aging consistently improved the interactions of asphalt with water and with calcite at all water layer thicknesses according to *E*_water-asph_ and *E*_asph-agg,res_, respectively. This improvement was attributable to the electronegativity of the added oxygens and thus the increased molecular polarity.

**Figure 12:**
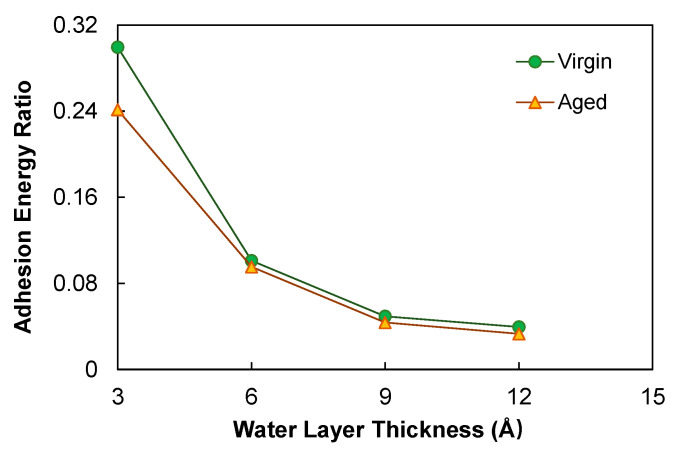
Adhesion energy ratio for the wet condition.

A comparison of the adhesion energies before and after water penetration in terms of the *E*_asph-agg,res_/*E*_asph-agg_ ratio was proposed as an indicator for the moisture susceptibility of the asphalt, as shown in Figure 12. The aged asphalt yielded lower ratios at all the four severity levels. This simulation result led to the inference that oxidation rendered the asphalt more sensitivity to moisture damage, which agrees with the general observations in the asphalt pavement industry.

### 3.4. Further Inspection of Increased Molecular Polarity

**Figure 13:**
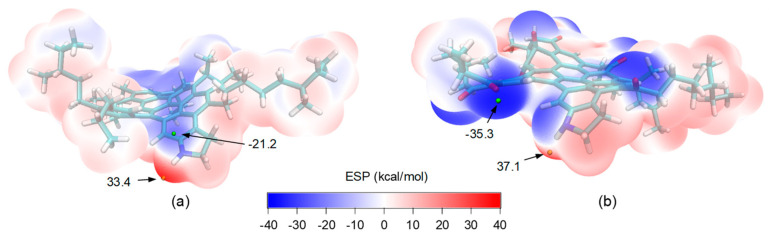
Electrostatic potential surfaces for (**a**) virgin and (**b**) aged asphaltene molecules.

A review of the preceding investigations revealed that an increase in the molecular polarity played a critical role in nearly all the aging-induced changes of the evaluated asphalt properties. The elevated polarity was largely responsible for the enhanced intermolecular attractiveness and weakened molecular mobility in the bulk asphalt, and also is the cause for the increased asphalt–aggregate interactions in both the dry and wet conditions. It is, therefore, of great importance and interest to further look into the specific impacts of aging on the molecular polarity. The asphaltene molecules were selected for this purpose since this fraction mostly dominates the engineering properties of asphalt and also presents the highest chemical reactivity with oxygen out of the four fractions. The electrostatic potential (ESP) surfaces for the virgin and aged asphaltenes were determined, which allowed for visualizing the three-dimensional charge distributions of the molecules. The computational method is given in the Appendix A. Figure 13 presents the obtained ESP surfaces, and maximal and minimal ESP points.

As shown in Figure 13a, for the virgin asphaltene molecule, the negative ESP was mostly concentrated at the polyaromatic core due to the π-electrons. The minimum with a value of −21.2 kcal/mol appeared in the vicinity of nitrogen from the pyrrole structure, owing to its lone electron pair. In the aged asphaltene as shown in Figure 13b, the oxygen atoms introduced at the benzyl carbons in the periphery of the polyaromatic core affected the distribution of π-electrons as a result of the strong electronegativity of oxygen and its two lone pairs of electrons. Hence, the local ESP minima were all located around the oxygens, in addition to the pyrrole nitrogen. The global minimum was found around a carbonyl with a value of −35.3 kcal/mol, which was considerably higher than that in the virgin case. The positive ESP was mostly located in the vicinity of the hydrogen atoms. For the virgin asphaltene, the maximal ESP was 33.4 kcal/mol, located along the extension of the N–H bond due to the electronegativity of nitrogen. In the aged case, the maximal ESP appeared at the same location, but was increased to 37.1 kcal/mol.

In addition to the ESP surface visualizing the spatial distribution of charges, the dipole moment was also determined for the asphaltene molecules, defined as

(4) $$\mu =\underset{j}{\sum }q_{j}x_{j}$$

where ***μ*** is the dipole moment vector, and *q_j_* and ***x****_j_* are the magnitude and position vector of the *j*-th charge, respectively. The dipole moment provides a measure of the overall polarity of a molecule. As expected, the aged asphaltene yielded a dipole moment of 5.034 Debye, one order of magnitude higher than 0.419 Debye for the virgin molecule.

The above analysis clearly demonstrates that oxidation rendered the charge distribution in the asphaltene molecule much more rugged, extended the positive and negative ESP extremes, and eventually boosted the molecular polarity. Similar observations are expected for the resin and aromatic components. These aging impacts ultimately enhance the electrostatic contribution to molecular interactions within the bulk asphalt and at the asphalt–aggregate interface.

## 4. Conclusions

This study investigated the impacts of oxidative aging on asphalt in terms of the performance-related engineering properties obtained from the molecular dynamics simulation. The following conclusions can be drawn on the basis of the findings:

- The oxidative aging of asphalt raised the density (from 1.008 to 1.081 g/cm^3^), cohesive energy density (from 3.02 × 10^8^ to 3.49 × 10^8^ J/m^3^), and glass transition temperature (from 315 to 345 K). The overall molecular polarity was elevated upon aging, as reflected in the increased electrostatic contribution to CED. The increase in *T_g_* was attributed to the stronger intermolecular attractiveness and reduced molecular mobility, which lowers the resistance of asphalt to thermal cracking.
- Aging considerably slowed down the diffusion process (and consequently the self-healing capacity) of asphalt at a given temperature. The diffusion behaviors of both the virgin and aged asphalt systems at different temperatures were well captured by the Arrhenius relationship, in which prefactor *D*_0_ was a valid index representing the overall effect of aging on asphalt diffusion. Oxidation substantially decreased the prefactor from 1.1 × 10^−5^ to 6.8 × 10^−6^ cm^2^/s.
- Aging benefited the adhesion between asphalt and calcite substrate in the dry condition, as it substantially improved the electrostatic interactions at the interface due to the increased molecular polarity. In the wet condition, the use of the proposed index, i.e., the ratio of the residual asphalt–aggregate adhesion to that in the dry condition, indicated that aging yielded consistently higher moisture susceptibility. The simulation results suggest that aging would increase the propensity of asphalt pavement to moisture damage.

The oxygen introduced into the molecules during oxidation significantly affected the charge distributions and ESP extremes. The resulting rise in molecular polarity was highly responsible for the aging-induced changes in the bulk and adhesive properties of asphalt. It can be inferred that, for the purpose of effectively reusing the oxidized asphalts, restoring the aging effects might be approached by mitigating the molecular polarity, which will be explored in future work.

## Figures and Tables

**Table 1:** Compositions of the virgin and aged asphalt models.

Components	Virgin			Aged		
	Formula	No. Ratio	Mass Ratio	Formula	No. Ratio	Mass Ratio
Asphaltene–pyrrole (asphaltenes)	C_66_H_81_N	2	18.2%	C_66_H_67_NO_7_	3	25.4%
Quinolinohopane (resins)	C_40_H_59_N	6	34.0%	C_40_H_55_NO_2_	7	35.0%
PHPN (aromatics)	C_35_H_44_	8	38.0%	C_35_H_36_O_4_	7	31.3%
Hopane (saturates)	C_35_H_62_	2	9.9%	C_35_H_62_	2	8.3%

## Data Availability

The data that support the findings also form part of an on-going study, and will be available from the corresponding author on request.

## Appendix A

The electrostatic potential was calculated on the basis of the Multiwfn code [45,46], and its visualization was accomplished via VMD [47]. The wavefunctions required as input into the code were obtained using Orca 5.0 software [48] with hybrid density functional *ω*B97M-V [49]. This functional was expected to provide better accuracy than the commonly used B3LYP with dispersion correction (i.e., B3LYP-D3) [50,51,52]. Triple-*ζ* basis set def2-TZVP was used in calculation [53].

## References

[1] Polacco G., Filippi S., Merusi F., Stastna G. (2015). A review of the fundamentals of polymer-modified asphalts: Asphalt/polymer interactions and principles of compatibility. Adv. Colloid Interface Sci..

[2] Christensen D.W., Bahia H.U., Anderson D.A. (1996). Effects of free volume and intermolecular friction on the viscosity of asphalt binders. J. Assoc. Asph. Paving Technol..

[3] Cao W. (2020). General fractional models for linear viscoelastic characterization of asphalt cements. J. Rheol..

[4] Cao W., Barghabany P., Mohammad L., Cooper S.B., Balamurugan S. (2019). Chemical and rheological evaluation of asphalts incorporating RAP/RAS binders and warm-mix technologies in relation to crack resistance. Constr. Build. Mater..

[5] Wang C., Chen Y., Cao W. (2019). A chemo-rheological approach to the healing characteristics of asphalt binders under short- and long-term oxidative aging. Constr. Build. Mater..

[6] Molenaar A., Hagos E., van de Vev M. (2010). Effects of aging on the mechanical characteristics of bituminous binders in PAC. J. Mater. Civil. Eng..

[7] Petersen J.C. (1998). A dual, sequential mechanism for the oxidation of petroleum asphalts. Pet. Sci. Technol..

[8] Petersen J.C., Harnsberger P.M. (1998). Asphalt aging: A dual oxidation mechanism and its interrelationships with asphalt composition and oxidative age hardening. Transp. Res. Rec..

[9] Petersen J.C., Glaser R. (2011). Asphalt oxidation mechanisms and the role of oxidation products on age hardening revisited. Road Mater. Pavement Des..

[10] Pan T., Lu Y., Lloyd S. (2012). Quantum-chemistry study of asphalt oxidative aging: An XPS-aided analysis. Ind. Eng. Chem. Res..

[11] Li D.D., Greenfield M.L. (2014). Chemical compositions of improved model asphalt systems for molecular simulations. Fuel.

[12] Li D.D., Greenfield M.L. (2014). Viscosity, relaxation time, and dynamics within a model asphalt of larger molecules. J. Chem. Phys..

[13] Pan J., Tarefder R.A. (2016). Investigation of asphalt aging behaviour due to oxidation using molecular dynamics simulation. Mol. Simul..

[14] Ding H., Wang H., Qu X., Varveri A., Gao J. (2021). Towards an understanding of diffusion mechanism of bio-rejuvenators in aged asphalt binder through molecular dynamics simulation. J. Clean. Prod..

[15] Gao Y., Zhang Y., Yang Y., Zhang J., Gu F. (2019). Molecular dynamics investigation of interfacial adhesion between oxidized bitumen and mineral surfaces. Appl. Surf. Sci..

[16] Qu X., Liu Q., Guo M., Wang D., Oeser M. (2018). Study on the effect of aging on physical properties of asphalt binder from a microscale perspective. Constr. Build. Mater..

[17] Xu G., Wang H. (2017). Molecular dynamics study of oxidative aging effect on asphalt binder properties. Fuel.

[18] Bhasin A., Little D.N., Vasconcelos K.L., Masad E. (2007). Surface free energy to identify moisture sensitivity of materials for asphalt mixes. Transp. Res. Rec..

[19] Alvarez A.E., Ovalles E., Caro S. (2012). Assessment of the effect of mineral filler on asphalt-aggregate interfaces based on thermodynamic properties. Constr. Build. Mater..

[20] Luo L., Chu L., Fwa T.F. (2021). Molecular dynamics analysis of oxidative aging effects on thermodynamic and interfacial bonding properties of asphalt mixtures. Constr. Build. Mater..

[21] Yao H., Dai Q., You Z. (2015). Chemo-physical analysis and molecular dynamics (MD) simulation of moisture susceptibility of nano hydrated lime modified asphalt mixtures. Constr. Build. Mater..

[22] Aguiar-Moya J.P., Salazar-Delgado J., Baldi-Sevilla A., Leiva-Villacorta F., Loria-Salazar L. (2015). Effect of aging on adhesion properties of asphalt mixtures with the use of bitumen bond strength and surface energy measurement tests. Transp. Res. Rec..

[23] Dassault Systèmes (2019). BIOVIA Materials Studio.

[24] Sun H., Jin Z., Yang C., Akkermans R.L.C., Robertson S.H., Spenley N.A., Miller S., Todd S.M. (2016). COMPASS II: Extended coverage for polymer and drug-like molecule databases. J. Mol. Model..

[25] King W.H., Corbett L.W. (1969). Relative oxygen absorption and volatility properties of submicron films of asphalt using the quartzite crystal microbalance. Anal. Chem..

[26] Lemarchand C.A., Schrøder T.B., Dyre J.C., Hansen J.S. (2013). Cooee bitumen: Chemical aging. J. Chem. Phys..

[27] Hartman P., Bennema P. (1980). The attachment energy as a habit controlling factor: I. Theoretical considerations. J. Cryst. Growth.

[28] Donnay J.D.H., Harker D. (1937). A new law of crystal morphology extending the law of Bravais. Am. Mineral..

[29] Stipp S.L.S., Eggleston C.M., Nielsen B.S. (1994). Calcite surface structure observed at microtopographic and molecular scales with atomic force microscopy (AFM). Geochim. Cosmochim. Acta.

[30] Herman A., Addadi L., Weiner S. (1988). Interactions of sea-urchin skeleton macromolecules with growing calcite crystals—A study of intracrystalline proteins. Nature.

[31] Wang P., Dong Z., Tan Y., Liu Z. (2015). Investigating the interactions of the saturate, aromatic, resin, and asphaltene four fractions in asphalt binders by molecular simulations. Energy Fuels.

[32] Tabatabaee H.A., Velasquez R., Bahia H.U. (2012). Modeling thermal stress in asphalt mixtures undergoing glass transition and physical hardening. Transp. Res. Rec..

[33] Anderson D.A., Marasteanu M.O. (1999). Physical hardening of asphalt binders relative to their glass transition temperatures. Transp. Res. Rec..

[34] Tabatabaee H.A., Velasquez R., Bahia H.U. (2012). Predicting low temperature physical hardening in asphalt binders. Constr. Build. Mater..

[35] Zhang L., Greenfield M.L. (2007). Analyzing properties of model asphalts using molecular simulation. Energy Fuels.

[36] Soldera A., Metatla N. (2006). Glass transition of polymers: Atomistic simulation versus experiments. Phys. Rev. E.

[37] Daly W.H., Negulescu I., Glover I. (2010). A Comparative Analysis of Modified Binders: Original Asphalts and Materials Extracted from Existing Pavements.

[38] Sun D., Sun G., Zhu X., Pang Q., Yu F., Lin T. (2017). Identification of wetting and molecular diffusion stages during self-healing process of asphalt binder via fluorescence microscope. Constr. Build. Mater..

[39] Sun D., Sun G., Zhu X., Ye F., Xu J. (2018). Intrinsic temperature sensitive self-healing character of asphalt binders based on molecular dynamics simulations. Fuel.

[40] Ding Y., Huang B., Shu X., Zhang Y., Woods M.E. (2016). Use of molecular dynamics to investigate diffusion between virgin and aged asphalt binders. Fuel.

[41] Xiao Y., Li C., Wan M., Zhou X., Wang Y., Wu S. (2017). Study of the diffusion of rejuvenators and its effect on aged bitumen binder. Appl. Sci..

[42] Tan Y., Guo M. (2013). Using surface free energy method to study the cohesion and adhesion of asphalt mastic. Constr. Build. Mater..

[43] Chu L., Luo L., Fwa T.F. (2019). Effects of aggregate mineral surface anisotropy on asphalt-aggregate interfacial bonding using molecular dynamics (MD) simulation. Constr. Build. Mater..

[44] Cucalon L.G., Kassem E., Little D.N., Masad E. (2017). Fundamental evaluation of moisture damage in warm-mix asphalts. Road Mater. Pavement Des..

[45] Lu T., Chen F. (2012). Multiwfn: A multifunctional wavefunction analyzer. J. Comput. Chem..

[46] Zhang J., Lu T. (2021). Efficient evaluation of electrostatic potential with computerized optimized code. Phys. Chem. Chem. Phys..

[47] Humphrey W., Dalke A., Schulten K. (1996). VMD—Visual molecular dynamics. J. Mol. Graph..

[48] Neese F., Wennmohs F., Becker U., Riplinger C. (2020). The ORCA quantum chemistry program package. J. Chem. Phys..

[49] Mardirossian N., Head-Gordon M. (2016). *ω*B97M-V: A combinatorially optimized, range-separated hybrid, meta-GGA density functional with VV10 nonlocal correlation. J. Chem. Phys..

[50] Becke A.D. (1993). Density-functional thermochemistry. III. The role of exact exchange. J. Chem. Phys..

[51] Lee C., Yang W., Parr R.G. (1988). Development of the Colle-Salvetti correlation-energy formula into a functional of the electron density. Phys. Rev. B.

[52] Grimme S., Antony J., Ehrlich S., Krieg H. (2010). A consistent and accurate ab initio parametrization of density functional dispersion correction (DFT-D) for the 94 elements H-Pu. J. Chem. Phys..

[53] Weigend F., Ahlrichs R. (2005). Balanced basis sets of split valence, triple zeta valence and quadruple zeta valence quality for H to Rn: Design and assessment of accuracy. Phys. Chem. Chem. Phys..

